# Fasting Neurotensin Levels in Pediatric Celiac Disease Compared with a Control Cohort

**DOI:** 10.1155/2020/1670479

**Published:** 2020-02-22

**Authors:** Donatella Iorfida, Monica Montuori, Chiara Maria Trovato, Claudio Tiberti, Andrea Sansone, Salvatore Cucchiara, Francesco Valitutti

**Affiliations:** ^1^Pediatric Gastroenterology and Liver Unit, Department of Maternal-Child Health and Urology, Sapienza University of Rome, Italy; ^2^Department of Experimental Medicine, Sapienza University of Rome, Italy; ^3^Center for Reproductive Medicine and Andrology, Universitätsklinikum Münster, Münster, Germany; ^4^Pediatric Unit, AOU San Giovanni di Dio e Ruggi d'Aragona, Salerno, Italy; ^5^EBRIS (European Biomedical Research Institute of Salerno), Salerno, Italy

## Abstract

**Background and Aims:**

Neurotensin (NT) is a gut hormone secreted by specific endocrine cells scattered throughout the epithelial layer of the small intestine, which has been identified as an important mediator in several gastrointestinal functions and disease conditions. Its potential involvement in celiac disease (CD) has been investigated, but there are conflicting findings. The aim of this study was to evaluate serum NT levels in children with CD at diagnosis, compared to a control group, and to investigate whether NT correlated in CD patients with symptoms, antibody response, and intestinal mucosal damage. *Materials and Methods*. Children (1-16 years old) undergoing gastrointestinal endoscopy for CD or for other clinical reasons were included in this study. Patients with CD diagnosed according to the 2012 European Society for Paediatric Gastroenterology, Hepatology and Nutrition (ESPGHAN) guidelines without biopsy were also recruited. Fasting serum samples were analyzed for NT levels using ELISA. Logistic regression, Wilcoxon rank sum, and Spearman's rank tests were used for statistical analysis.

**Results:**

Thirty children (18 females, 2.2-15.9 years old) were enrolled. Of 25 patients who underwent endoscopy, 9 were CD patients, 13 were controls, and 3 were excluded due to nonspecific inflammation at duodenal biopsy. CD was diagnosed in 5 patients without biopsy. NT median was higher in CD patients compared to controls (13.25 (IQR 9.4-17.5) pg/ml vs. 7.8 (IQR 7.6-10) pg/ml; *p* = 0.02). No statistically significant association between NT and clinical, serological, or histological data of CD was observed in this CD cohort.

**Conclusions:**

To our knowledge, this is the first study that evaluates NT in CD children from Italy. Results show that NT is higher in the serum of CD children at diagnosis compared to controls. However, larger-scale studies are required to validate these findings. Whether serum NT levels can be an adjunctive marker for pediatric CD remains currently elusive.

## 1. Introduction

Neurotensin (NT) is a 13-amino acid peptide first isolated in 1973 from the bovine hypothalamus and digestive tract [1]. Its physiological functions are those of neurotransmitter in the central nervous system and hormone in the periphery.

Centrally, it affects sensory and motor functions, temperature regulation, neuroendocrine control of the pituitary, and control of blood flow and pressure [2].

In the gut, it is secreted by endocrine N cells scattered predominantly in the epithelial layer of the jejuno-ileum and released after a meal, particularly those containing high lipid levels [3]. It has a range of paracrine and endocrine functions regulating gastrointestinal secretion and motility under physiological conditions [2, 4].

Thus, NT has been shown to play an important role in the conduction of multiple physiologic processes in both the brain and the periphery. Disruption of these normal mechanisms may contribute to the development of various diseases [5].

Previous studies have demonstrated that alterations of NT levels were associated with several neuropathological conditions [6] and have supported the role of NT in endocrine, autocrine, and paracrine growth stimulation of several types of cancer [5].

NT might also participate in various pathophysiological gastrointestinal processes, including the modulation of intestinal responses to stressful and inflammatory stimuli that share several common features such as mast cell and immune cell activation. By interacting with specific receptors, NT exerts direct and indirect effects on nerves, epithelial cells, and cells of the immune and inflammatory systems [4], an aspect that has captured scientific attention in the last three decades.

It would be fascinating to explore aspects that are still little known regarding the involvement of this peptide in gastrointestinal diseases, and experimental research towards this direction should be supported.

Among gastrointestinal diseases, celiac disease (CD) is in the foreground for its notable social burden [7] and its features of systemic condition affecting several organs [8]. CD presents a multifactorial etiopathogenesis, and despite many advances in terms of understanding the disease's mechanisms in recent years, many aspects remain to be defined.

CD is a systemic, immune-mediated disorder triggered by gluten and related prolamines in genetically susceptible individuals. It encompasses the presence of a variable combination of clinical manifestations, CD-specific antibodies, human leukocyte antigen- (HLA-) DQ2 or HLA-DQ8 haplotypes, and immune-mediated enteropathy [9].

Onset can occur at any age, and although the inflammatory process specifically targets the intestinal mucosa, patients may present with gastrointestinal signs or symptoms, extraintestinal signs or symptoms, or both [10]. Nevertheless, some patients display only minor clinical features or even no symptoms at diagnosis [11].

Antibodies against tissue transglutaminase (anti-tTG), an endogenous protein, are highly sensitive and used as specific markers for CD. However, in some pediatric cases and all adult cases, an intestinal biopsy is required to confirm the diagnosis [12].

The histological examination allows to identify different degrees of intestinal inflammation and villous atrophy, which correlates with levels of anti-tTG. In support of this, several studies confirmed that high concentration of anti-tTG in serum predicts villous atrophy better than low or borderline values [9, 13].

A potential involvement of NT in CD has been investigated, but there are conflicting findings.

In 1978, Bloom et al. showed that patients with untreated CD had an increase in postprandial NT levels compared to healthy controls and CD subjects on a gluten-free diet [14]. In that year, the same group of authors assessed the profile of intestinal hormones in CD patients, showing in these subjects an increase in fasting NT levels [15]. However, these data were not confirmed in a pediatric study a few years later [16]. In 2000, Bardella et al. identified increased fasting NT levels in CD patients paralleled by a reduced postprandial rebound compared to controls [17].

Recently, the goal of a pediatric study on CD patients has been to assess whether NT could be a spy for more important forms of intestinal inflammation [18]. In particular, Montén et al. have dosed the proneurotensin precursor fragment 1-117, referred to as proneurotensin (pro-NT), shown to be completely stable in human plasma and to be produced in equimolar amounts with respect to NT [19]. The correlation between pro-NT and severity of CD clinical picture was assessed with regard to antibody titers and histological damage. They found that plasma pro-NT levels were elevated in children with CD and in those with severe intestinal mucosal damage, hypothesizing that pro-NT could play a role in small intestinal inflammation.

Following the path of this latest research, the aim of our study was to evaluate serum NT levels in children with CD at diagnosis compared to a control group. A further aim was to investigate whether NT correlated in CD patients with symptoms, antibody response, and intestinal mucosal damage.

## 2. Materials and Methods

The study was conducted between November 2017 and May 2018 at the Pediatric Gastroenterology and Liver Unit of Policlinico Umberto I, Sapienza, University of Rome, Italy. The local ethical committee approved the study. All participants were informed about the aim of the study, and a parental written consent was obtained for each child.

### 2.1. Patient Selection

Children (1-16 years old) who underwent gastrointestinal endoscopy for CD or for other clinical indications (i.e., unexplained anemia, poor growth, dysphagia, heartburn, and epigastric pain) were included in the study. CD patients diagnosed according to the 2012 ESPGHAN guidelines [9] without biopsy were also recruited.

Exclusion criteria were gluten-free diet, neoplasia, immunosuppressive therapy, neurological/neuropsychiatric pathology, history of allergy/mastocytosis, and history of intestinal infection/inflammation (i.e., recent infectious gastroenteritis, recent fever episode, inflammatory bowel diseases, eosinophilic esophagitis, and eosinophilic gastroenteropathy).

### 2.2. Blood Sampling, Gastrointestinal Endoscopy, and Diagnostic Classification

All patients enrolled were fasting prior to blood sampling for at least six hours for toddlers (≤2 years) and at least nine hours for children older than 2. If endoscopy was performed, the blood sampling was taken at the same time as the procedure. All serum samples were analyzed for NT and screened for CD (anti-tTG IgA and total serum IgA). HLA typing and endomysial antibodies (EMA) were performed when required for diagnosis according to the 2012 ESPGHAN guidelines.

The same pathologist scored all biopsies according to Marsh-Oberhuber (MO) criteria, in a blinded way with regard to the results of CD screening and clinical data.

Children who resulted positive for anti-tTG and showing grading 2 or 3 according to MO classification were defined as having CD. Anti-tTG-negative children with negative duodenal biopsy (i.e., without any kind of histopathological alterations and with less than 25 intraepithelial lymphocytes/100 enterocytes) were included as disease controls. Subjects with histopathologic signs of nonspecific inflammation or features attributable to other diseases in duodenal biopsies were excluded from the study.

Children positive for anti‐tTG levels ≥ 10 times the upper limit of normal (ULN), EMA IgA, and HLA DQ2 and/or DQ8 with suggestive symptoms were defined as having CD without biopsy, according to the 2012 ESPGHAN guidelines.

### 2.3. CD Screening

Fasting serum samples were analyzed for anti-tTG levels using ELISA (Eurospital, Trieste, Italy). Values ≥ 16 UA/ml were considered positive results.

The dosage of serum total IgA, if not already available, was performed in each patient to rule out an IgA deficiency. The amount of serum IgA was measured by nephelometry.

When required for diagnosis, EMA were performed through indirect immunofluorescence (Eurospital, Trieste, Italy), whereas for HLA typing, a molecular biology system (Eu-Gen System, Eurospital, Trieste, Italy) was used.

### 2.4. Neurotensin Analysis

For quantitative determination of serum NT levels, the commercial Human Neurotensin ELISA was used (catalog number ABIN365746 on antibodies-online.com). Blood samples were collected using a serum separator tube and were centrifuged according to the manufacturer's instructions. Serum was removed, aliquoted, and stored at -80°C to avoid loss of bioactivity. After thawing the samples, serum NT levels were determined. The detection range of the kit was 15.6-1000 pg/ml. Samples were analyzed at the Department of Experimental Medicine, Sapienza, University of Rome.

### 2.5. Clinical Evaluation of Patients

For each child, body weight, height, and body mass index were recorded, and a detailed history was collected in order to define the presence of intestinal and/or extraintestinal symptoms/signs of CD.

### 2.6. Statistical Analysis

Logistic regression, Wilcoxon rank sum, and Spearman's rank tests were used for statistical analysis (R software). A *p* value < 0.05 was considered significant for all the tests performed. NT levels were log-transformed due to their skewed distribution.

## 3. Results

### 3.1. Patient Diagnosis

Thirty children (18 F; 2.2-15.9 years old) were enrolled in this study. CD was diagnosed in 14 patients (10 F; mean age 6.6 years), 13 were recruited as controls (8 F; mean age 12.1 years), and 3 patients were excluded due to nonspecific inflammation at duodenal biopsy. CD was diagnosed in 5 of 14 patients without biopsy, according to the 2012 ESPGHAN guidelines (Table 1).

Histology of all CD patients showed villous atrophy (MO grading: 3) (Table 1). None were found to have potential CD. Duodenum biopsies of all controls were negative, and only mild superficial chronic gastritis or mild reflux esophagitis was found at histology in this group (Table 2). These latter findings were observed also in some CD patients (2/9) (Table 1).

Among patients with CD, 7 had intestinal and extraintestinal symptoms of CD, 2 had no symptoms suggestive of CD, and in the remaining 5 patients, symptoms were purely intestinal or extraintestinal. As regards anti-tTG values, 7 of them had titers ≥ 10 times the ULN: among these, 2 had no symptoms suggestive of CD, so the no-biopsy option was not applied for diagnosis.

### 3.2. Neurotensin Results

**Figure 1:**
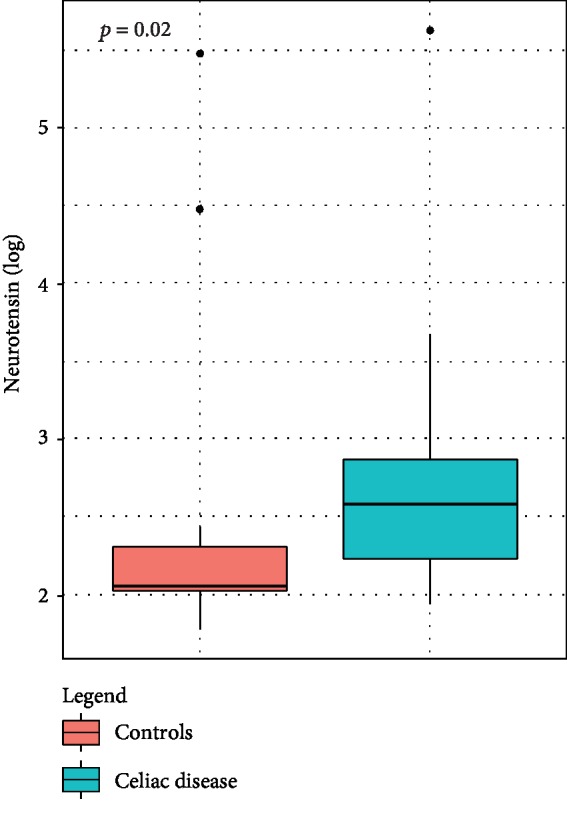
Fasting serum levels of neurotensin in CD patients and controls. The horizontal line indicates the medians (log-transformed scale) in a box and whisker plot (*p* = 0.02).

NT median was higher in CD patients compared to controls (13.25 (IQR 9.4-17.5) pg/ml vs. 7.8 (IQR 7.6-10) pg/ml; *p* = 0.02) (Figure 1). Nevertheless, no statistically significant correlation of NT with clinical presentation of CD, anti-tTG titers, and mucosal damage graded according to MO criteria was observed in this cohort.

Moreover, no correlation was observed between NT and age within each of the two groups and among all the overall cohort of twenty-seven patients considered for NT analysis.

## 4. Discussion

In our study, we found that children with untreated CD had increased fasting serum NT levels compared to controls.

In line with our results, increased fasting NT levels were detected in two previous studies in adult CD patients: Besterman et al. have found in CD patients an increase in fasting NT levels [15], and the Italian group of Bardella et al. have reported similar evidence [17]. These data, however, were not confirmed in a pediatric study from 1987 [16].

Recently, Montén et al. have found elevated peripheral pro-NT levels to reflect more severe forms of active CD in children [18]. In this study, pro-NT levels were measured in plasma by a chemiluminometric sandwich immunoassay to detect a pro-NT precursor fragment, based on an assay described by Ernst et al. [19]. Fasting pro-NT levels were found higher in children with CD compared to the disease controls. An association was observed between the anti-tTG and pro-NT levels in plasma. Furthermore, plasma pro-NT levels in children with MO 3b and MO 3c histology were higher than those in children with MO 0 [18].

Released pro-NT ideally represents NT as they have been shown to circulate in equimolar amounts [19].

In our cohort, no statistically significant correlation of NT with serological or histological data of CD was observed. In particular, we have not found any statistically significant correlation of NT with anti-tTG levels or MO score, although it is likely that the small sample size influenced these results. None of the enrolled patients had a MO score < 3, so we could not unveil any correlation between NT and different degrees of intestinal mucosal damage.

In our study, we also looked for a potential relationship between NT and clinical presentation of CD.

The high rate of dyspepsia-like symptoms frequently reported in CD patients may be related to upper gastrointestinal tract motor abnormalities [20–23]. The pathophysiology of these motor abnormalities may involve gastrointestinal hormones, the secretion of which may be altered in CD patients as a consequence of intestinal mucosal damage [15, 16, 24]. Regarding a potential role of NT, Bardella et al. [17] have found not only that untreated CD patients had significantly higher fasting plasma NT levels than controls but also that the baseline NT levels in both groups correlated significantly with the gastric emptying time, thus suggesting that NT may physiologically inhibit upper gastrointestinal motility.

Nevertheless, no association between NT levels and intestinal and/or extraintestinal symptoms was observed in our cohort, although a relationship with specifically dyspepsia-like symptoms was not investigated. It should be highlighted that NT is one of several hormones that modulate gastric emptying and intestinal motility.

With regard to CD symptoms, the frequent coexistence of irritable bowel syndrome (IBS) and CD [25] is also food for thought.

Some indirect data suggest the potential involvement of NT in the pathophysiology of IBS. Many factors related to the exacerbation of symptoms in IBS, such as psychiatric disorders, certain foods, and intestinal infections, influence the central or peripheral secretion of NT [26]. Moreover, mechanisms involved in this syndrome, like gut dysmotility, altered brain–gut axis, stress-related enteric responses, visceral hypersensitivity, microbial flora overgrowth, low-grade intestinal inflammation, and mast cell hyperreactivity, could be partially explained by dysfunction in NT pathway [26]. Furthermore, NT influences the molecular circuits of other peptides with a proven role in IBS [26].

It could be speculated that the frequent IBS-like syndrome observed in CD patients could be partially explained by a raised NT level, but further research to sustain this assumption is needed. However, this aspect was not investigated in our work, requiring obviously a larger-scale study.

It is notable that in our cohort, age was a confounder for CD, with increased frequency of this condition among young children: mean age of our CD patients was 6.6 years, whereas that of controls was 12.1 years. However, no correlation was observed between NT and age among patients enrolled, differently from the study of Montén et al. [18]. These authors have found an impact of age on pro-NT levels among controls with a tendency to lower levels among older children. Nevertheless, in their cohort, no correlation was noticed between age and pro-NT among children with CD, so the impact of age on pro-NT levels seemed irrelevant compared with the impact of age on CD diagnosis.

A limitation of our study is the small number of patients enrolled: this makes the chances of finding statistically significant associations very low. Moreover, the relatively large number of patients who have received biopsy-sparing CD diagnosis limits further the ability to correlate NT with the severity of histological damage.

Another limitation of our work could be that the disease controls were children investigated for various reasons. Access to reference levels for fasting NT levels in both young and older healthy children would be required, but it is not feasible from an ethical point of view.

The strength of this research was the enrollment of control subjects with clear absence of intestinal inflammation as shown by small bowel biopsy. Patients with history of intestinal infection/inflammation, with histopathologic signs of nonspecific inflammation, or features attributable to other diseases in duodenal biopsies were rigorously excluded from our study. The only histological abnormalities considered acceptable were mild superficial chronic gastritis or mild reflux esophagitis, outcomes that were also found in some CD patients (2/9). Moreover, in order to avoid any confounding factors, all subjects enrolled in this study were previously selected according to medical history to exclude any allergic, inflammatory, neoplastic, or neurologic conditions, in light of the possible involvement of NT in these diseases [27, 28].

Nevertheless, it is of note that for controls, no bulbar biopsy was obtained, differently from CD patients. Albeit this could theoretically be a bias, it is improbable that patients without serology and duodenal histology for CD host an inflammatory lesion in the duodenal bulb. However, a sufficient number of duodenal biopsies were taken altogether for controls (at least three in the second and third portions of the duodenum as per hospital endoscopy protocol). Thus, we are quite confident that, even though a small bias might be introduced to this regard, it is very unlikely that it undermines results and conclusions. Furthermore, there was no clinical indication for bulbar biopsy in control patients at the time of the procedure.

For discussion purposes, it is important to consider that NT and its receptors can be localized in both the central nervous system and along the length of the gastrointestinal tract. As NT can directly activate immune, inflammatory, epithelial, and neuronal cells, the mechanisms by which this peptide triggers many diverse intestinal responses may be difficult to dissect. It is conceivable that conflicting findings regarding its involvement in intestinal conditions such as CD are to be partly attributed to this complex network [4]. The mechanisms governing the altered NT levels in CD patients are still unclear, although it has been suggested that this finding may be related to a compensatory increase in NT secretion from specific endocrine cells of the unaffected ileal mucosa [17]. Chronic small intestine mucosal inflammation leading to endocrine cell dysfunction and motor abnormalities in CD patients may also be implicated [21]. Thus, in order to clarify whether the increase in NT levels may represent an additional diagnostic marker for CD, further research is needed, especially with regard to underlying mechanisms that are poorly defined.

In addition, normal levels of NT have been reported by Besterman et al. [15] in CD patients on a gluten-free diet, and this data could suggest a potential role of NT also as a disease follow-up marker. Anyway, this evaluation was beyond the aim of our study, and for this purpose, a different study design would be appropriate.

An additional aspect to mention is that in recent years, pro-NT, the stable NT precursor fragment in human blood, has gained attention and its role has been investigated in different conditions [29]. A clinical study showed that plasma pro-NT is released in equimolar amounts similar to NT in circulation under physiological conditions [19] and it was found possibly converted into an active NT. However, there is no further evidence to support this point. In light of this consideration, although Montén et al. in a recent pediatric study [18] have suggested a potential role of pro-NT in CD, we aimed to evaluate a potential involvement of NT, echoing previous studies [14–17]. It is unquestionable that it would be interesting to evaluate both NT and pro-NT at the same time, albeit it was not feasible in our study.

## 5. Conclusions

To our knowledge, this is the first Italian study that evaluates NT in pediatric CD patients. These results have shown that NT is higher in the serum of CD children at diagnosis compared to controls. However, larger-scale studies are required to validate these findings and to allow further speculations on this issue. So far, whether serum NT levels can be an adjunctive marker for pediatric CD remains currently elusive.

## Figures and Tables

**Table 1:** This table illustrates clinical presentation, endoscopic, and histological findings for each enrolled CD patient. In the column “No-biopsy approach,” CD patients diagnosed according to the 2012 ESPGHAN guidelines without biopsy are indicated.

CD patient	Gender (M/F)	Age (years)	Clinical presentation	Endoscopic findings	Histological findings	No-biopsy approach^∗^
CD patient 1	M	6.1	Constipation, recurrent abdominal pain, fatigue, impaired growth, anemia	No-biopsy protocol has been adopted^∗^	No-biopsy protocol has been adopted^∗^	X
CD patient 2	F	3.6	Constipation	Irregular surface of duodenal folds	MO grading 3b. H. pylori-negative chronic superficial gastritis, mild reflux esophagitis	
CD patient 3	F	4.6	Recurrent abdominal pain, fatigue	Mild edema and hyperemia of the duodenal bulb and duodenum, reduced height and irregular surface of duodenal folds	MO grading 3b	
CD patient 4	M	3.1	Constipation, oral aphthosis, recurrent abdominal pain, astenia	No-biopsy protocol has been adopted^∗^	No-biopsy protocol has been adopted^∗^	X
CD patient 5	F	10.9	Meteorism, abdominal pain, tooth enamel defects, first-degree relative of the CD patient	No-biopsy protocol has been adopted^∗^	No-biopsy protocol has been adopted^∗^	X
CD patient 6	F	6.3	Impaired growth	Mild hyperemia of the duodenal bulb	MO grading 3a. Mild reflux esophagitis	
CD patient 7	M	2.2	Lack of appetite, fatigue, impaired growth	No-biopsy protocol has been adopted^∗^	No-biopsy protocol has been adopted^∗^	X
CD patient 8	F	7.7	No symptoms (screening)	Negative	MO grading 3b-3c	
CD patient 9	F	7.5	Recurrent abdominal pain	Negative	MO grading 3b	
CD patient 10	F	3.7	Lack of appetite, fatigue, impaired growth	Mild edema of the duodenal bulb	MO grading 3b-3c	
CD patient 11	F	13	Constipation, lack of appetite, recurrent abdominal pain, fatigue, anemia, tooth enamel defects	No-biopsy protocol has been adopted^∗^	No-biopsy protocol has been adopted^∗^	X
CD patient 12	F	6.5	No symptoms (screening)	Mild edema and hyperemia of the duodenal bulb, reduced height, and irregular surface of duodenal folds	MO grading 3b-3c	
CD patient 13	M	10.7	Dental enamel hypoplasia	Mild edema and hyperemia of the duodenal bulb, irregular surface of duodenal folds	MO grading 3c	
CD patient 14	F	6.6	Constipation, recurrent abdominal pain	Mild edema and hyperemia of duodenum, reduced height, and irregular surface of duodenal folds	MO grading 3c	

^∗^According to the 2012 ESPGHAN guidelines: symptoms suggestive of CD, serum anti‐tTG IgA levels ≥ 10 times the ULN, positive EMA-IgA, positive CD HLA risk alleles DQ2 and/or DQ8.

**Table 2:** This table illustrates, for each enrolled control, the clinical indication for endoscopy, endoscopic and histological findings, and related diagnosis.

Controls	Gender (M/F)	Age (years)	Clinical indication for endoscopy	Endoscopic findings	Histological findings	Diagnosis
Control 1	F	13.7	Vomiting blood	Antral nodularity	H. pylori-negative chronic superficial gastritis	H. pylori-negative chronic superficial gastritis
Control 2	F	15.9	Vomiting	Negative	Negative	Functional disorder
Control 3	F	13.6	Epigastric pain	Cardias incontinence	H. pylori-negative chronic superficial gastritis, mild reflux esophagitis	H. pylori-negative chronic superficial gastritis, mild reflux esophagitis
Control 4	F	14.9	Gastroesophageal reflux symptoms, abdominal pain, vomiting	Longitudinal striae in the distal esophagus	Mild reflux esophagitis	Mild reflux esophagitis
Control 5	F	12.7	Recurrent abdominal pain	Negative	Mild reflux esophagitis	Mild reflux esophagitis
Control 6	F	15.7	Epigastric pain, gastroesophageal reflux symptoms	Negative	Mild reflux esophagitis	Mild reflux esophagitis
Control 7	M	8.6	Chest pain, gastroesophageal reflux symptoms	White specks in the distal esophageal mucosa	H. pylori-negative chronic superficial gastritis	H. pylori-negative chronic superficial gastritis, esophagitis
Control 8	F	15.8	Epigastric pain	Negative	H. pylori-negative chronic superficial gastritis	H. pylori-negative chronic superficial gastritis
Control 9	M	11.4	Epigastric pain, vomiting	Mild hyperemia third distal esophagus, cardias incontinence	Mild reflux esophagitis	Mild reflux esophagitis
Control 10	M	14.7	Poor growth, lack of appetite	Negative	Negative	Poor growth
Control 11	F	2.7	Epigastric pain vomiting	Duodenal bulb nodularity	Mild reflux esophagitis	Mild reflux esophagitis
Control 12	M	8.1	Vomiting	Inlet patch	Inlet patch, mild reflux esophagitis	Inlet patch, mild reflux esophagitis
Control 13	M	8.4	Recurrent vomiting	Longitudinal striae in the distal esophagus	Mild reflux esophagitis	Mild reflux esophagitis

## Data Availability

The data used to support the findings of this study are available from the corresponding author upon request.
